# Machine learning for predicting clinical outcomes of hospitalised children: a systematic review of applications in low- and middle-income countries

**DOI:** 10.1016/j.eclinm.2025.103743

**Published:** 2026-01-08

**Authors:** William Nkhono, Eva van Lieshout, Job Calis, Violet Naanyu, Mark Hoogendoorn, Kamija S. Phiri, María Villalobos-Quesada

**Affiliations:** a Amsterdam Institute for Global Health and Development (AIGHD), Amsterdam, the Netherlands; b Department of Computer Science, Vrije Universiteit Amsterdam (VU), the Netherlands; c Kamuzu University of Health Science (KUHES), Blantyre, Malawi; d Training and Research Unit of Excellence (TRUE), Malawi; e Department of Paediatric Intensive Care, Emma Children's Hospital, Amsterdam UMC, Meibergdreef, the Netherlands; f Department of Paediatrics and Child Health, Kamuzu University of Health Sciences, Malawi; g School of Arts and Social Sciences Department, Moi University, Kenya; h National eHealth Living Lab (NeLL), Department Public Health and Primary Care, Leiden University Medical Centre, the Netherlands

**Keywords:** Artificial intelligence, Machine learning, Systematic review, Children, Hospitalised, Low- and middle-income countries

## Abstract

**Background:**

Machine Learning (ML) can contribute to reducing child mortality and morbidity in low- and middle-income countries (LMICs), yet its development and clinical adoption remain unclear. This systematic review provides an overview of ML for hospitalised children in LMICs.

**Methods:**

In June 2025, searches in five scientific databases and one scholarly search engine identified 26 eligible peer-reviewed studies using ML on hospitalised children under 18. Studies using only conventional statistics and perinatal data were excluded. Study quality and bias were assessed using PROBAST + AI. Descriptive statistics were used for data analysis. PRISMA reporting guideline was followed.

**Findings:**

These studies were conducted in Asia (58%) and Sub-Saharan Africa (38%), mostly retrospective (62%), and predominantly used patient files (62%). The median sample size was 1291. Prognostic models dominated (69%), primarily targeting mortality (50%). Ensemble methods were most common (50%). The median AUROC was 0.81 (IQR 0.78–0.83). Most models were at a clinical Readiness Level 3–4 (81%). Barriers and facilitators related to data (65%, 34% respectively), implementation (50%, 77%), technology (31%, 42%), and human (19%, 35%) were reported.

**Interpretation:**

We provided evidence of ML's promising performance for LMICs. Mortality prediction was the main focus. Arriving at clinical applications that benefit LMICs, requires investment in high-quality data and alignment to local (clinical) needs.

**Funding:**

This project is part of the EDCTP2 programme (grant number RIA2020I-3294 IMPALA) supported by the European Union.

**Research in context**

**Evidence before this study**

Studies of ML algorithms for health and healthcare have been increasing in high-income countries (HICs) and low- and middle-income countries (LMICs). However, the biggest proportion of scientific publications still comes from HICs. The development and final clinical adoption of ML in LMICs face challenges hard to overcome, such as a lack of digital infrastructure, high-quality data, skilled personnel, and a data-driven culture. Nevertheless, researchers in LMICs are making valuable contributions to the field. We searched Google Scholar, PubMed, Embase, Scopus, Web of Science and IEEE Xplore for scientific literature from database inception in October 2024 to June 2025, for papers published with no language restrictions, using the terms related to Machine Learning (ML), hospitalisation and children, with a focus on studies that apply ML to solve challenges specific to LMICs and their populations, aiming to improve access and the provision of high-quality care. Our search yielded 2737 reports.

One challenge of LMICs that cannot remain unsolved is the high rate of child mortality and morbidity. Children constitute a vulnerable population whose needs should be prioritised and this is necessary to achieve the desired universal health coverage.

**Added value of this study**

This study makes visible the efforts to develop and use ML for paediatric in-hospital care in LMICs. It offers a critical overview of the ML tools being developed and their characteristics, providing the opportunity to understand the global landscape of these tools, and helping to identify key areas necessary to prioritise to accelerate the clinical application of ML.

**Implications of all the available evidence**

Although ML holds immense potential to transform the care of hospitalised children in LMICs and models show promising predictive power according to their stage of development, ML tools still have to transition efficiently to the bedside. The readiness level of the included studies was comparable to HIC. However, the limitation of resources in LMIC, such as the lack of pre-existing data-related infrastructures, poses additional barriers to transition to clinical applications. Investing in digital infrastructure for providing care, and research and innovation in LMICs is necessary. As models become more mature, ensuring the ML's clinical relevance and building adequate evidence to determine the value of ML models in clinical practice becomes more pressing. Ethical and legal considerations such as data privacy, algorithmic bias and transparency must remain central to ML.

## Introduction

More than five million children under the age of five died globally in 2020 from preventable causes, with over 80% of these deaths occurring in low- and middle-income countries (LMICs) in sub-Saharan Africa (SSA) and southern Asia.^1^ For children in these regions, and in LMICs in general, access to high-quality healthcare, including in-hospital care,^2^ remains a challenge due to limitations in timely diagnosis and effective treatment, and the acutely scarce infrastructure and medical staff.^3^

Machine learning (ML),^4^ a subdomain of artificial intelligence (AI), focuses on algorithms capable of learning from experience to improve performance at specific tasks without being explicitly programmed.^5^ This technique is a promising data-driven approach that can address challenges in LMICs and improve access to high-quality care.^6^ For instance, by leveraging available data, ML has the potential to enhance diagnostic time and accuracy where specialist expertise is scarce, for example, detecting birth asphyxia from infant cries,^7^ or identifying diabetic retinopathy.^8^ In oncology, ML has been applied to optimise the allocation of available tests and treatments where resources are limited.^9^ Additionally, ML has been proposed as a (partial) solution to mitigate workforce shortages, either by translating expert knowledge to local staff (for example, via smartphone-based applications), or supporting task-shifting (where non-specialists perform tasks typically reserved for specialists).^10^^,^^11^ Overall, these applications can support clinical decision-making, improving in turn, patient outcomes.^12^ In the case of infectious disease management, ML has been used to model and predict the occurrence of diseases such as dengue,^13^ thereby aiding resource allocation and preparedness within healthcare systems. Such applications can have particularly impactful effects in LMICs where the burden of preventable deaths remains high.^11^^,^^14^

Scientific research on ML is dominated by studies on populations in high-income countries (HIC).^15^ However, the perceived potential of ML is triggering advances in LMICs. The number of publications from LMICs on ML is rising, including applications in hospital settings.^15^ However, translating this research into routine clinical practice remains limited, similar to what is observed in HICs.^12^ Potential challenges observed in HICs, such as data scarcity, data quality, and concerns about transparency, explainability, bias, and negative effects on the work floor,^5^ may overlap with those in LMICs. However, challenges in LMICs are yet to be comprehensively studied.

Although ML could contribute to addressing specific challenges in LMICs, there is currently no overview of how the ML field is advancing in this setting. Additionally, comprehensive and systematic studies about potential barriers and facilitators in the development and implementation of ML models in routine clinical practice are missing. This systematic review contributes to filling that gap by providing a systematic overview of the current ML approaches used for hospitalised children to improve healthcare outcomes in low-resource settings (LRS) within LMICs.

## Methods

### Search strategy and selection criteria

We addressed the following research question: What ML approaches have been applied to improve health outcomes for hospitalised children in LRS within LMICs, and which barriers and facilitators have been reported for their development and implementation in clinical practice?

We focused on ML approaches developed for hospitalised children in LRS and within LMICs as defined by the World Bank.^16^^,^^17^ We defined LRS in the healthcare domain, as a setting characterised by limited access and availability to medication, equipment, supplies, devices, underdeveloped infrastructure and limited trained personnel.^18^

Two researchers (W.N., E.L.) systematically searched Google Scholar, PubMed, Embase, Scopus, Web of Science and IEEE Xplore for scientific literature in June 2025, without applying any date restrictions. A free-text search was carried out, based on the following string: (“artificial intelligence” OR “machine learning”) (“hospitalised” OR “admission” OR “clinical outcome”) (child∗ OR infant OR paediatric) (“low resource setting” OR “low middle-income countries”).

Studies that met the following inclusion criteria were included: (1) the study developed, tested, or applied ML techniques, (2) the study population was hospitalised children under 18 in LMICs, and (3) publications were peer-reviewed full-text original research. Studies were excluded if they exclusively employed conventional statistics. In order to distinguish ML models from conventional statistics models, we adopted the definition proposed by van Boven et al., where ML is defined as a data-driven approach, without manual selection of features and/or inclusion of more complex models such as neural networks.^19^ Additionally, studies were excluded when focused solely on perinatal data.^20^ We did not restrict the inclusion to any particular study design, language or timeframe. However, searches were carried out exclusively in English. In cases where a study developed multiple models, we reported the best-performing model.

Identified articles were exported to Rayyan AI,^21^ where duplicates were automatically identified and manually verified. Titles, abstracts and full texts were reviewed independently by W.N. and E.L., and discrepancies were resolved through discussion with a third reviewer (J.C). To ensure accuracy and validity, all extracted data were subsequently verified by M.V. Any remaining clinical questions were addressed to J.C., and ML questions towards M.H. The recommendations outlined in the Preferred Reporting Items for Systematic Reviews and Meta-analyses (PRISMA) were followed.^22^ The review was not registered in PROSPERO.

### Study risk of bias assessment

We evaluated the quality, risk of bias and applicability of each study using PROBAST + AI framework, a tool designed for healthcare prediction models, whether based on regression or AI techniques.^23^ The assessment was carried out W.N and J.C verified a proportion of the studies. Any discrepancies or unsure studies flagged by W.N were resolved via discussion within the research team.

### Data analysis

Included studies were characterised according to (1) authors, (2) title, (3) year of publication, (4) region of the study population, (5) study design, (6) sample size, (7) clinical application, (8) outcome, (9) data source and (10) type of data as defined by Annis et al.^24^

ML approaches were characterised according to (1) type of ML approach,^25^ (2) performance metrics, and (3) clinical readiness level (RL).^4^ The clinical RL classification was specifically developed for ML applications in intensive care, and is applicable to other hospital care settings. It comprises the following levels: (RL1) identification of the clinical problem, (RL2) proposal of model/solution, (RL3-4) model prototyping and development, (RL5) model validation, (RL6) real-time model testing, (RL7) workflow implementation, (RL8) clinical outcome evaluation, and (RL9) model integration in clinical practice.

Barriers and facilitators were categorised based on an adaptation of the themes reported by Ahmed et al., 2023^26^: (1) data-related such as quality, acquisition and incomplete datasets, (2) computing and physical infrastructure such as servers, software, buildings, data collection machines or sensors, (3) human such as stakeholders, skills, training, (4) ethical and legal such as compliance and data privacy and (5) implementation such as clinical relevance, transparency, explainability or user acceptance.

Due to the heterogeneity of the included studies regarding clinical settings, types of data and endpoints, a meta-analysis was not feasible. Data were analysed using descriptive statistics.

### Ethics statement

No formal ethical approval and informed consent were required for this review, as it involved secondary analysis of publicly available data and did not involve the collection of new data from human participants.

### Role of the funding source

This project is part of the EDCTP2 programme (grant number RIA2020I-3294 IMPALA)^27^ supported by the European Union. The funder played no role in study design, data collection, data analysis, data interpretation, or manuscript writing.

## Results

**Fig. 1:**
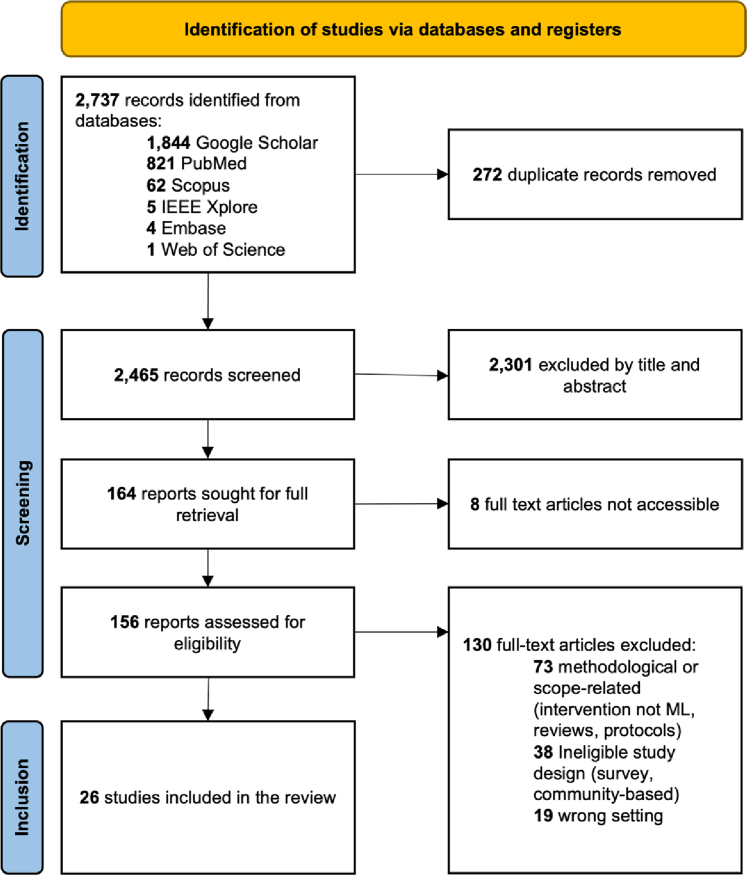
Study selection of evidence using Preferred Reporting Items for Systematic Reviews and Meta-Analyses for systematic review guidelines (PRISMA) flow diagram.^22^

Our search identified 2737 studies, and 272 duplicates were removed. After screening titles and abstracts, 164 articles remained for full-text screening, of which 156 could be retrieved. Finally, 26 studies, published between 2017 and 2024, met the inclusion criteria (Fig. 1).

Out of the 26 studies, fifteen (58%) were conducted in Asia,^28^, ^29^, ^30^, ^31^, ^32^, ^33^, ^34^, ^35^, ^36^, ^37^, ^38^, ^39^, ^40^, ^41^, ^42^ ten (38%) in Sub-Saharan Africa (SSA)^43^, ^44^, ^45^, ^46^, ^47^, ^48^, ^49^, ^50^, ^51^, ^52^ and one (4%) in both Asia and SSA.^53^ Sixteen (62%) were retrospective studies,^29^^,^^31^^,^^32^^,^^34^, ^35^, ^36^, ^37^, ^38^, ^39^^,^^41^, ^42^, ^43^^,^^46^^,^^48^^,^^49^^,^^52^ eight (31%) were prospective,^28^^,^^30^^,^^33^^,^^44^^,^^45^^,^^47^^,^^50^^,^^53^ and two (8%) used both retrospective and prospective designs^40^^,^^51^ (Table 1).

**Table 1:** Characteristics of included studies.

First author	Title	Year	Region	Study design	Sample size	Clinical application	Outcome	Data source	Type of data
Ming	Applied machine learning for the risk-stratification and clinical decision support of hospitalised patients with dengue in Vietnam	2022	Asia	Retros	4131	Diag	Dengue shock syndrome	Specialised research database	Patient demographics, Patient clinical observation, Laboratory data
Tran	A simple nomogram to predict dengue shock syndrome	2024	Asia	Retros	4522	Diag	Dengue shock syndrome	Patient files	Patient demographics, Patient clinical observation
Tunthanathip	Comparison of intracranial injury predictability between machine learning algorithms and the nomogram in paediatric traumatic brain injury	2021	SSA	Retros & pros	964	Diag	Intracranial injury	Patient files	Patient demographics, Patient health-related behaviours and social history, Patient clinical observation, Radiology imaging
Babenko	Ability of Procalcitonin and C-Reactive Protein for Discriminating between Bacterial and Enteroviral Meningitis in Children Using Decision Tree	2021	Asia	Pros	269	Diag	Meningitis	Patient files	Patient demographics, Patient clinical observation, Laboratory data
Hwang	Machine learning-based prediction of critical illness in children visiting the emergency department	2022	Asia	Retros	2,621,710	Prog	Critical illness	Patient files	Patient demographics, Patient health-related behaviours and social history, Patient clinical observation, Healthcare costs and expenditures
Lee	Prediction of hospitalisation using artificial intelligence for urgent patients in the emergency department	2021	Asia	Retros	282,971	Prog	Hospitalisation	Patient files	Patient demographics, Patient clinical observation
Das	Development of machine learning models predicting mortality using routinely collected observational health data from 0 to 59 months old children admitted to an intensive care unit in Bangladesh: critical role of biochemistry and haematology data	2024	Asia	Retros	3505	Prog	Mortality	Patient files	Patient demographics, Patient clinical observation, Laboratory data
CHAIN network	Characterising paediatric mortality during and after acute illness in SSA and South Asia: a secondary analysis of the CHAIN cohort using a machine learning approach	2023	SSA and Asia	Pros	3101	Prog	Mortality	Patient files	Patient demographics, Patient health-related behaviours and social history, Patient clinical observation
Genisca	Constructing, validating, and updating machine learning models to predict survival in children with Ebola Virus Disease Constructing, validating, and updating machine learning models to predict survival in children with Ebola Virus Disease	2022	SSA	Retros	579	Prog	Mortality	Patient files	Patient demographics, Patient health-related behaviours and social history, Patient clinical observation, Laboratory data
Hsu	Machine learning approaches to predict in-hospital mortality among neonates with clinically suspected sepsis in the neonatal intensive care unit	2021	Asia	Retros	1095	Prog	Mortality	Patient files	Patient demographics, Patient clinical observation, Laboratory data
Kovacs	Developing practical clinical tools for predicting neonatal mortality at a neonatal intensive care unit in Tanzania	2021	SSA	Pros	165	Prog	Mortality	Specialised research database	Patient demographics, Patient health-related behaviours and social history, Patient clinical observation
Kwizera	A Machine Learning Based Triage Tool for Children with Acute Infection in a Low Resource Setting	2019	SSA	Pros	1579	Prog	Mortality	Specialised research database	Patient demographics, Patient health-related behaviours and social history, Patient clinical observation
Pienaar	An Artificial Neural Network Model for Paediatric Mortality Prediction in Two Tertiary Paediatric Intensive Care Units in South Africa. A Development Study	2022	SSA	Retros	2089	Prog	Mortality	Specialised research database	Patient demographics, Patient clinical observation, Laboratory data
Pienaar	Development of artificial neural network models for paediatric critical illness in South Africa	2022	SSA	Pros	765	Prog	Mortality	Specialised research database	Patient demographics, Patient clinical observation
Domíngez-Rodríguez	Machine learning outperformed logistic regression classification even with limited sample size: A model to predict paediatric HIV mortality and clinical progression to AIDS	2022	SSA	Pros	100	Prog	Mortality	Specialised research database	Patient demographics, Patient clinical observation, Laboratory data
Sheikhtaheri	Prediction of neonatal deaths in NICUs: development and validation of machine learning models	2021	Asia	Retros & Pros	1762	Prog	Mortality	Specialised research database	Patient demographics, Patient clinical observation
Tuti	An exploration of mortality risk factors in non-severe pneumonia in children using clinical data from Kenya	2017	SSA	Retros	10,687	Prog	Mortality	Specialised research database	Patient demographics, Patient clinical observation
Lin	Machine learning models to evaluate mortality in paediatric patients with pneumonia in the intensive care unit	2024	Asia	Retros	1231	Prog	Mortality	Patient files	Patient demographics, Patient clinical observation, Laboratory data
Robi	Neonatal Disease Prediction Using Machine Learning Techniques	2023	SSA	Retros	2298	Prog	Sepsis, birth asphyxia, necrotising enterocolitis, and respiratory distress syndrome	Patient files	Patient demographics, Patient clinical observation, Laboratory data, Radiology imaging
Rahimi	A Preliminary Investigation into Use of Admission-Recorded Photoplethysmograms for Predicting Hospital Mortality in Children with Confirmed or Suspected Infection in Resource-Poor Settings	2023	SSA	Retros	6533	Prog	Mortality	Patient files	Patient demographics, Patient clinical observation, Laboratory data
Liu	A novel combined nomogram for predicting severe acute lower respiratory tract infection in children hospitalised for RSV infection during the post-COVID-19 period.	2024	Asia	Retros	1351	Prog	Severe acute lower respiratory tract infection	Patient files	Patient demographics, Patient clinical observation, Laboratory data
Garbern	A novel digital health approach to improving global paediatric sepsis care in Bangladesh using wearable technology and machine learning	2022	Asia	Pros	100	Prog	Sepsis	Specialised research database	Patient demographics, Patient clinical observation, Laboratory data, Radiology Imaging
Kanwal	Diagnosis of Community-Acquired pneumonia in children using photoplethysmography and Machine learning-based classifier	2024	Asia	Pros	67	Diag	Community-Acquired Pneumonia	Specialised research database	Patient demographics, Patient clinical observation, Laboratory data
Oonsivilai	Using machine learning to guide targeted and locally-tailored empiric antibiotic prescribing in a children's hospital in Cambodia	2018	Asia	Retros	243	Therag	Antibiotic susceptibility	Patient files	Patient demographics, Patient health-related behaviours and social history, Patient clinical observation
Xue	Machine learning for screening and predicting the risk of anti-MDA5 antibody in juvenile dermatomyositis children	2022	Asia	Retros	152	Therag	Screening anti-MDA5 antibodies	Patient files	Patient demographics, Patient clinical observation, Laboratory data
Kashef	Prediction of Cranial Radiotherapy Treatment in Paediatric Acute Lymphoblastic Leukaemia Patients Using Machine Learning: A Case Study at MAHAK Hospital	2020	Asia	Retros	241	Therag	Cranial radiotherapy	Patient files	Patient demographics, Patient clinical observation, Laboratory data

Diag, Diagnostic; Prog, Prognostic; Pros, Prospective; Retros, Retrospective; Therag, Theragnostic; SSA, Sub-Saharan Africa.

Studies were ordered by clinical application and outcome.

The median sample size was 1291 patients (IQR 250–3155). Eight studies (31%) had a sample size between 60 and 500 patients,^28^^,^^30^^,^^33^^,^^34^^,^^39^^,^^42^^,^^44^^,^^50^ three (12%) 501–1000 patients,^43^^,^^47^^,^^51^ eleven (42%) 1001–5000 patients^29^^,^^31^^,^^36^, ^37^, ^38^^,^^40^^,^^41^^,^^45^^,^^46^^,^^49^^,^^53^ and four (15%) more than 5000^32^^,^^35^^,^^48^^,^^52^ (Table 1).

Prognostic clinical applications dominated, reported in eighteen studies (69%).^29^, ^30^, ^31^, ^32^^,^^35^, ^36^, ^37^^,^^40^^,^^43^, ^44^, ^45^, ^46^, ^47^, ^48^, ^49^, ^50^^,^^52^^,^^53^ Among these, thirteen studies (72%) mainly focused on mortality, mortality-associated risk factors and survival. The remaining prognostic applications focused on hospitalisation,^35^ sepsis, birth asphyxia, necrotising enterocolitis, and respiratory distress syndrome,^30^^,^^49^ critical illness,^32^ and severe acute lower respiratory tract infection.^37^ Diagnostic and theragnostic ML models were less common. Five diagnostic models (19%) aimed to diagnose meningitis,^28^ intracranial injury,^51^ community acquired pneumonia^33^ and dengue shock syndrome.^38^^,^^41^ Three theragnostic models (12%) developed predictions for screening anti-MDA5 antibodies in dermatomyositis patients,^42^ guided antibiotic prescription^39^ and predicted if Acute Lymphoblastic Leukaemia patients needed cranial radiotherapy.^34^

Types of data were categorised according to the framework by Annis et al.^24^ Patient demographics and clinical observations were the most frequently used data types reported in all 26 studies (100%). Laboratory data were used in thirteen studies (50%),^28^, ^29^, ^30^, ^31^^,^^34^^,^^36^, ^37^, ^38^^,^^42^^,^^43^^,^^46^^,^^49^^,^^50^ health-related behaviours and social history were used in seven studies (27%),^32^^,^^39^^,^^43^, ^44^, ^45^^,^^51^^,^^53^ radiographic data in three studies (12%)^30^^,^^49^^,^^51^ and healthcare cost and expenditure data in one study (4%).^32^

Data sources were categorised as patient files or specialised research databases. Patient files were defined as records generated and maintained for routine patient care and used primarily for clinical purposes such as diagnosis, treatment, monitoring, and communication among healthcare providers. This included structured paper case report forms that were digitised. Patient files were used in sixteen studies (62%).^28^^,^^29^^,^^31^^,^^32^^,^^34^, ^35^, ^36^, ^37^^,^^39^^,^^41^, ^42^, ^43^^,^^48^^,^^49^^,^^51^^,^^53^ Specialised research databases, a combination of routine care and research data, contain data extracted from patient files and often incorporate additional data elements not routinely collected in clinical practice. These databases, sometimes built using tools like REDCap, were employed in ten studies (38%).^30^^,^^33^^,^^38^^,^^40^^,^^44^, ^45^, ^46^, ^47^^,^^50^^,^^52^

Ensemble methods were the most commonly used ML approach reported in thirteen studies (50%).^29^^,^^32^^,^^34^^,^^36^^,^^39^^,^^42^^,^^43^^,^^45^^,^^48^, ^49^, ^50^, ^51^^,^^53^ Within this category, Random Forest, reported in eight studies (62%), was the most used ML technique, followed by Stacking models in two studies (15%),^34^^,^^49^ XGBoost in two studies (15%)^36^^,^^53^ and Elastic Net Model in one study (8%).^43^ The second most frequent ML approach was Neural Networks, used in six studies (23%),^31^^,^^35^^,^^38^^,^^40^^,^^46^^,^^47^ this category included five studies (83%) that applied Artificial Neural Networks (ANN) and one (17%) that applied Deep Neural Networks (DNN).^31^ The third category was learning linear models, used in five studies (19%), specifically Partial Least Squares – Discriminant Analysis (PLS-DA),^52^ LASSO regression,^37^ logistic regression,^41^ Ridge regression^30^ and Generalised Linear Models (GLMs).^44^ Classification regression tree methods (4%), specifically Fast and Frugal Trees^28^ and Instance-based learning (4%) with a weighted K-nearest neighbour,^33^ were used in one study each.

The performance evaluation metrics reported in the studies showed promising results, with various metrics used to assess effectiveness (Table 2). Twenty-two studies (85%) reported more than one metric. The area under the receiver operating characteristic (AUROC) was reported in twenty-three studies (88%) and was the most common metric, with a median value of 0.81 (IQR 0.78–0.83), indicating good discriminative ability.^29^, ^30^, ^31^, ^32^, ^33^, ^34^, ^35^, ^36^, ^37^, ^38^, ^39^, ^40^, ^41^, ^42^, ^43^, ^44^, ^45^, ^46^, ^47^^,^^50^, ^51^, ^52^, ^53^ Among these, thirteen studies (57%) focused on predicting mortality, achieving AUROC values ranging from 0.73 to 0.92. AUROC values above 0.80 suggest potential clinical feasibility.

**Table 2:** Performance of ML models, readiness level and barriers, and facilitators.

First author	ML approach^a^		Metrics						RL 1–9^b^	Barriers	Facilitators
	ML category^a^	ML technique	AUROC	Spec	Sens	Acc (%)	F1	AUPRC			
Babenko	Classification and Regression Tree	Fast and Frugal Trees		0.96	1.00	98			RL3-4	Data	Implementation
Genisca	Ensemble Methods	Elastic Net Model	0.77						RL5	Data, Human	Implementation
Domíngez-Rodríguez	Ensemble Methods	Random Forest	0.73		0.78	83			RL3-4	Implementation	Human
Kwizera	Ensemble Methods	Random Forest	0.79			81			RL3-4	Data, Human	Data, Computing, Human, Implementation
Oonsivilai	Ensemble Methods	Random Forest	0.80						RL3-4	Computing	Implementation
Tunthanathip	Ensemble Methods	Random Forest	0.80	0.34	0.95	79			RL6	Data, Implementation	Human, Implementation
Das	Ensemble Methods	Random Forest	0.85						RL3-4	Data	Data, Computing
Xue	Ensemble Methods	Random Forest	0.97						RL3-4	Computing, Implementation	Implementation
Hwang	Ensemble Methods	Random Forest	0.99					0.64	RL3-4	Data, Implementation	Data
Kashef	Ensemble Methods	Stacking model (Gradient Boosting Machine, Distributed Random Forest)	0.87			90			RL3-4	Data, Computing	Computing, Implementation
Robi	Ensemble Methods	Stacking model (Support Vector Machine, Random Forest, XGBoost)		0.97	0.97	97	0.97		RL3-4	Computing	Implementation
Lin	Ensemble Methods	XGBoost	0.80	0.71	0.71	69	0.19	0.15	RL3-4	Data, Implementation	Implementation
CHAIN network	Ensemble Methods	XGBoost	0.83	0.88	0.66				RL3-4	Computing, Human	Computing, Human
Kovacs	Learning linear models	Generalised Linear Models	0.76	0.68	0.76				RL3-4	Data, Computing	Implementation
Liu	Learning linear models	LASSO regression	0.82	0.76	0.78				RL5	Data	Computing, Human, Implementation
Tran	Learning Linear Models	Logistic Regression	0.85	0.84	0.71	82			RL3-4	Implementation	Data, Implementation
Tuti	Learning linear models	Partial Least Squares - Discriminant Analysis	0.76						RL3-4	Human, Ethical and Legal	Data, Computing
Lee	Neural Networks	Artificial Neural Network	0.80	0.78	0.67				RL3-4	Data, Human, Implementation	Data, Implementation
Ming	Neural Networks	Artificial Neural Network	0.83	0.88	0.66				RL5	Data, Implementation	Computing, Implementation
Pienaar	Neural Networks	Artificial Neural Network	0.89					0.60	RL3-4	Data, Computing, Implementation	Computing, Human
Sheikhtaheri	Neural Networks	Artificial Neural Network	0.92	0.83	0.86	86	0.91		RL6	Data	Implementation
Pienaar	Neural Networks	Artificial Neural Network	0.84					0.64	RL3-4	Implementation	Data, human, Implementation
Hsu	Neural Networks	Deep Neural Network	0.92	0.83	0.97	96	0.77		RL3-4	Implementation	Computing, Implementation
Garbern	Learning linear models	Ridge regression	0.86	0.75	0.83	76	0.53		RL3-4	Data, Computing, Implementation	Data, Computing, Human and Implementation
Kanwal	Instance-based learning	Weighted K-nearest Neighbour	0.75	0.69	0.80				RL3-4	Data	Implementation
Rahimi	Ensemble Methods	Random Forest		0.71	0.95				RL3-4	Data, Implementation	Data, Computing, Human, Implementation

Studies were ordered according to ML category.

Acc, Accuracy; AUPRC, Area Under the Precision-Recall Curve; AUROC, Area Under the Receiver Operating Characteristic Curve; F1, F1 Score; Sens, Sensitivity; Spec, Specificity.

a ML category according to ACM's computing classification system (CCS)[19].

b Clinical Readiness Level (RL) [20]: RL1 identification of the clinical problem, RL2 proposal of model/solution. RL3-4 model prototyping and development, RL5 model validation, RL6 real-time model testing, RL7 workflow implementation, RL8 clinical outcome evaluation, RL9 model integration in clinical practice.

Other metrics, such as specificity and sensitivity, were reported in fifteen studies (65%)^28^^,^^30^^,^^31^^,^^33^^,^^35^, ^36^, ^37^, ^38^^,^^40^^,^^41^^,^^44^^,^^48^^,^^49^^,^^51^^,^^53^ and one study (4%) reported sensitivity only.^50^ Specificity ranged from 0.34 to 0.97 and sensitivity from 0.66 to 1.00. Accuracy was reported in eleven studies (42%),^28^^,^^30^^,^^31^^,^^34^^,^^36^^,^^40^^,^^41^^,^^45^^,^^49^, ^50^, ^51^ from 69% to 98%. Five studies (19%)^30^^,^^31^^,^^36^^,^^40^^,^^49^ reported the F1 score ranging from 0.19 to 0.97. Four studies (15%) included the area under the precision–recall curve (AUPRC), ranging from 0.15 to 0.64.^32^^,^^36^^,^^46^^,^^47^ The significance of these metrics varies greatly depending on the specific study design, disease, clinical context, and the potential penalties of prediction errors. The variability of the included studies makes it impossible to establish universal acceptable performance thresholds or to directly compare metrics across studies that involve different populations, outcomes, and designs. However, the metrics reported are important as an indication of the models' performance and predictive capabilities. Reporting multiple relevant metrics demonstrates a thorough approach to evaluation.

Models' clinical readiness level (RL) ranged from RL3 to RL6 (Table 2). Twenty-one models (81%) were at RL3-4 (model prototyping and development).^28^, ^29^, ^30^, ^31^, ^32^, ^33^, ^34^, ^35^, ^36^^,^^39^^,^^41^^,^^42^^,^^44^, ^45^, ^46^, ^47^, ^48^, ^49^, ^50^^,^^52^^,^^53^ These studies showed the potential of ML models to predict or assist clinical decisions or to be further optimised and validated on characterised datasets.^4^ Three models (12%) were at RL5 (model validation).^37^^,^^38^^,^^43^ These studies focused on testing and evaluating the ML models using realistic datasets other than the original training and testing population to ensure their generalisability and reliability.^4^ Two models (8%) were at RL6 (real-time model testing). Sheikhtaheri et al. used an Ensemble approach^40^ for predicting neonatal intensive care unit (NICU) deaths, and Tunthanathip used a Neural Network approach^51^ to predict intracranial injury. These studies evaluated model performance in real-time and integrated the models into the electronic health record or hospital system in one or more clinical settings, but there was no implementation into the clinical workflow, e.g. prospective observational studies comparing model performance in standard of care.^4^ There were no models beyond RL6.

All studies reported barriers and facilitators influencing the development and implementation of ML models in healthcare. These were categorised as (1) data-related, (2) computing and physical infrastructure, (3) human, (4) ethical and legal, and (5) implementation.^26^

Data barriers that hindered model development and potentially compromised the models' reliability were reported in seventeen studies (65%).^28^, ^29^, ^30^^,^^32^, ^33^, ^34^, ^35^, ^36^, ^37^, ^38^^,^^40^^,^^43^, ^44^, ^45^^,^^47^^,^^48^^,^^51^ These included difficulties with data quality, class imbalance, small sample size, and completeness. Conversely, existing infrastructure for paperless hospitals, electronic databases, generation of prospective data, large datasets, and relatively complete datasets acted as facilitators in nine studies (34%).^29^^,^^30^^,^^32^^,^^35^^,^^41^^,^^45^^,^^46^^,^^48^^,^^52^

Implementation barriers were reported in thirteen studies (50%),^30^, ^31^, ^32^^,^^35^^,^^36^^,^^38^^,^^41^^,^^42^^,^^46^, ^47^, ^48^^,^^50^^,^^51^ including the lack of external validation, generalisability, or insufficient evidence of cost-effectiveness. Facilitators of implementation were mentioned in twenty studies (77%),^28^^,^^30^^,^^31^^,^^33^, ^34^, ^35^, ^36^, ^37^, ^38^, ^39^, ^40^, ^41^, ^42^, ^43^, ^44^, ^45^, ^46^^,^^48^^,^^49^^,^^51^ including the use of SHapley Additive exPlanations (SHAP) method for explaining model output, use of simple models with simple predictors or models with high discriminatory ability.

Technological barriers related to physical infrastructure and hardware capabilities were reported in eight studies (31%),^30^^,^^34^^,^^39^^,^^42^^,^^44^^,^^47^^,^^49^^,^^53^ such as using paper-based files, and lack of electronic health records and digital data infrastructure. In contrast, the availability of advanced open-source software (e.g. Python, R, REDCap), existing infrastructure for paperless hospitals and potential of smartphone apps acted as facilitators in eleven studies (42%).^29^, ^30^, ^31^^,^^34^^,^^37^^,^^38^^,^^45^^,^^47^^,^^48^^,^^52^^,^^53^

Human barriers, including challenges related to user acceptance of prediction models, were present in five studies (19%).^35^^,^^43^^,^^45^^,^^52^^,^^53^ These included limited skills to interpret risk scores, indicating a need for training, as well as lack of model explainability (e.g. of black box models) and the associated negative impact on trust. Facilitators were identified in nine studies (35%),^30^^,^^37^^,^^45^, ^46^, ^47^, ^48^^,^^50^^,^^51^^,^^53^ such as multidisciplinary collaborations and the acceptance of models due to their perceived clinical relevance.

All studies had ethical and legal approval from an ethics board. One study (4%)^52^ reported reluctance to adopt international guidelines on pneumonia in SSA acted as a barrier. Other studies did not report legal or ethical barriers and facilitators.

The PROBAST + AI assessment (see Supplementary Appendix) indicated low concerns regarding the applicability of the included ML models, and most of them showed good performance. However, methodological rigour is a concern. The models' quality and generalisability was limited by small sample sizes, retrospective study designs, heterogeneous data sources, and selection bias. Risk of bias during evaluation highlighted a high concern due to the absence of a formal calibration assessment and net benefit reported in 6 studies (23%).^37^^,^^38^^,^^42^^,^^43^^,^^46^^,^^47^ Most models showed a strong discrimination AUC of 0.80 (IQR 0.78–0.83), but a few reported complementary performance metrics such as positive predictive value (PPV), limiting assessment of false positive rates. The limited evaluation metrics limited clinical applicability.

## Discussion

This review is, to our knowledge, the first to collate ML models for hospitalised children in LMICs. We found a limited number of studies (n = 26) from SSA and Asia. Studies were published after 2017, although no publication date criteria were implemented. The field is developing prognostic clinical applications, predicting mortality, using mainly ensemble methods and achieving good discriminatory ability. Most studies used retrospective data, consistent with their stage of development, as most models were at clinical RL3-4. Facilitators for developing and implementing ML models included: data and technology (e.g. access to digital data, infrastructure, and software), implementation (e.g. use of available explainability methods, simple predictors, and acceptable performance), human aspects (e.g. multidisciplinary teams and model's clinical relevance) and ethical and legal (e.g. reluctance to adopt new WHO pneumonia classification). Key barriers included data and technology (e.g. availability of high-quality data and infrastructure), implementation (e.g. lack of external validation, generalisability, and cost-effectiveness evidence), and human aspects (e.g. insufficient skills, limited explainability and lack of trust).

The use of ML to predict mortality and critical-care related outcomes in LRS responds to the clinical need for early recognition of patients at risk, providing healthcare personnel with the opportunity to intervene earlier and more efficiently, and save human lives.^54^ If successful, an ML approach can potentially facilitate efficient distribution of resources, both human and material. The value of ML compared to conventional statistics remains a topic of discussion, with evidence suggesting that ML models tend to perform better in “large N, small p” settings,^55^ including mortality prediction.^56^^,^^57^ They have also shown advantages in highly innovative fields with large, complex datasets such as omics, radiodiagnostics, drug development and personalised treatment.^58^ However, this highly innovative scenario may not match the reality in LMICs. ML approaches should tested and empirical evidence about their superiority over conventional statistics should be provided.

We also showed that researchers in LMICs are tackling other clinical needs with diagnostic and theragnostic models in fields such as trauma, cancer, vector-borne diseases, antibiotic sensitivity, leukaemia treatment, and rare diseases. These efforts can contribute in the future to addressing broader healthcare issues in LMICs,^59^ e.g. by compensating for a lack of infrastructure such as diagnostic facilities or specialised staff, such as radiologists^60^^,^^61^ and can contribute towards personalised medicine.^62^

This study also showed a clear preference for ensemble methods, a popular method in general.^63^ Ensemble methods are relatively simple and offer high interpretability, making them suitable for health applications. They require less computing power and have a low demand for smaller datasets than deep learning techniques, making them easier to develop, in the LRS of LMICs.^11^ The prominence of ensemble methods aligns with the use of other simpler yet effective ML techniques such as PLS-DA, LASSO, logistic regressions, and GLMs; methods also applied in the included studies. The focus on simple, interpretable models that healthcare providers can understand builds trust and increases adoption.^64^ However, deep learning techniques have their advantages. They can perform very well with complex data like medical images, yet we showed that this application is still limited, presumably because of the limited digital radiological data.^11^ This may change, as it has been observed in upper-middle-income countries.^63^

Many of the reported models are proof of concept tools at an RL3-4. Only 12% of studies have performed external validation, a critical step for assessing generalisability and robustness. This supports the reported gap between research and clinical use.^4^^,^^5^ Nevertheless, from a performance perspective, the models can be considered promising, showing, for example, reasonable AUROC (median 0.81, IQR 0.78–0.83). Consistent with the low clinical RL of the included studies, nearly two-thirds used retrospective data.^59^ Retrospective studies have lower risks and costs, and are easier to conduct compared to prospective studies. While retrospective studies serve as a valuable foundation,^61^ prospective studies are crucial for model clinical validation across various clinical settings.^65^ Prospective data collection, creating robust databases and improving data quality will lead to clinically useful ML applications.^66^ The slow shift to prospective data collection and validation delays the readiness of these models for real-world clinical use.^59^ To transition to more advanced clinical RLs, models need to address methodological shortcomings (such as small sample sizes, retrospective study design, heterogeneous data sources, and selection bias), and associated risks of bias and limited reporting of evaluation metrics. Additionally, the application of ML to address needs in LMIC should also raise the more fundamental question of whether ML is the right approach. Addressing, for example, the question of whether ML can objectively outperform traditional methods in the task at hand.

To facilitate the transition into higher RLs, and therefore ML's adoption in clinical practice, attention needs to be paid to LMICs' challenges. While HICs often encounter hurdles related to ethical and regulatory compliance, leading to slower integration into complex workflows,^67^ LMICs face more fundamental obstacles.^68^ Our results showed that data, implementation, and technological aspects were the main challenges in LMICs. Not that these challenges are absent in HIC,^69^^,^^70^ but they are acute in LMICs. Aiming at increasing the availability of high-quality data and the local necessary infrastructures (data and technical aspects) will only be successful if we invest in acquiring the necessary computer skills and AI literacy of researchers and end users (human aspects). Aspects such as user acceptability, integration into workflows, and economic sustainability can be expected to become more tangible the more advanced the RL. Facing clinical implementation will raise ethical questions and challenges, which will increase awareness for future ML applications.^71^ Although perceiving the development in the field of ML in such a linear way can help to simplify a complex situation, plenty of evidence in HIC indicates that failing to consider users (views, preference and needs) and ethical and legal aspects from the inception of ML application, will hinder its successful clinical application.^54^^,^^72^^,^^73^ Our results suggest that leveraging existing resources (material and human), prioritising clinically relevant, actionable and interpretable ML, and fostering collaboration can maximise the impact of ML in settings with limited resources.^65^

LMICs are advancing in the AI field, developing regulations and best practices, and conceptualising concepts such as trustworthy AI.^74^ As found in our study, regulatory uncertainty is a problem for developers, and working toward clear national and regional strategies can be part of the solution. These strategies should address technical, human, ethical, and legal aspects comprehensively.^75^ Our research shows that AI and ML development in LMIC is happening, albeit on a smaller scale than in HIC and it is addressing locally relevant needs. The 26 studies in our review, which focused on hospitalised children, show ML models with an acceptable performance for their stage or development, directly contradicting the idea that AI in LMICs is a “chimera”.^68^ In Table 3, we offer general recommendations based on the findings of this study.

**Table 3:** General recommendations for developing ML for Clinical adoption in LMIC.

Recommendation focus	Actionable steps and rationale
1. Think and design ahead	Map barriers and facilitators early in the ML development process for clinical use and apply them to draft a development plan.
2. Focus on data availability and quality	Address data and technological aspects immediately, such as investing in digital data collection and server capacity to improve data quality, and developing datasets for model development. This will help generate more and higher-quality data, reduce bias (or the risk of it), and improve model quality and generalisability.
3. Keep the clinical application and end user in mind	Advancing towards clinical adoption will depend on effectively incorporating users' perspectives into the design, development, validation, and implementation of ML models. Invest early in characterising user needs, acceptability, workflow integration, and economic sustainability.
4. Focus on clinical implementation	Prioritise addressing major LMIC health challenges as they will have the greatest impact. Report all necessary performance metrics, including those that are clinically relevant. Evaluate ML applications for clinical relevance and actionability, and prioritise implementation only when and where ML can provide improvements in patient care.
5. Keep it simple and explainable	Match ML methods to clinical needs and available resources. Use simple, interpretable models when explainability is required, as this would increase clinical uptake. Invest in making outputs explainable and actionable to end users, reserving complex techniques for cases where they bring clear added value.
6. Leapfrog while learning from HICs	Anticipate ethical and regulatory challenges as ML approaches clinical use. Design ML systems with compliance in mind and contribute proactively to best practices and regulations. Do not forget to build on the experiences of others, including in HICs.
7. Increase visibility and equitable collaboration	Establish equitable international networks (among LMICs, or between LMICs and HICs) to develop, validate, and share models and resources. Consider how your current research can contribute to lasting data infrastructures and local and regional AI strategies. If developers are based in non-English-speaking LMICs, consider publishing in both the local language and English to ensure international visibility.
8. Empower policy and governance actors	Policy-makers, regulatory bodies, ethics committees, funding agencies, and international organisations such as the WHO should prepare a facilitating environment for developing and integrating ML into healthcare in a safe, responsible, and effective way.

AI, Artificial Intelligence; LMIC, Low- and Middle-Income Country; HIC, High-Income Country; ML, Machine Learning; WHO, World Health Organisation.

To interpret this study, it is necessary to consider its limitations. We identified a limited number of studies; however, this is in line with previous work,^66^ highlighting a technology and knowledge gap.^76^ It is possible that ML tools for our specific clinical scope were not published in scientific literature, as it has been reported before,^77^ or not captured due to the English-language-based search strategy. Furthermore, our focus on hospitalised patients excluded ML studies with other health applications and could have emphasised mortality as an outcome. Including other populations might have yielded different results. We need to acknowledge that we could not control for publication bias. Lastly, the lack of studies from regions other than SSA and Asia shows the need for future research to assess the state of ML development in other parts of the world. We should not assume a lack of ML development in other regions, based solely on the absence of published data found in this review.

Our systematic analysis of the ML models for predicting clinical outcomes for hospitalised children in LMICs showed that, regardless of the challenges, Asian and African regions are contributing to the field of ML and to addressing healthcare challenges in these LRS. LMICs must prioritise technological development, including data infrastructure, capacity building, regulation, and policy. This is needed to achieve AI's full potential. This review contributes to amplifying the presence of LMICs in the field of ML, and offers an overview of barriers and facilitators of ML development that can inform funders, policymakers, researchers, and healthcare.

## Contributors

WN, JC, MH, KP and MV conceived the idea for this review. WN designed and wrote the review protocol with critical input from EL, JC, MH, KP, and MV. WN and EL developed the search strategy and conducted the searches in all databases. WN and EL independently screened all references and determined eligibility. JC oversaw and adjudicated the study selection process. WN extracted the data and EL and MV verified the data. WN drafted the first version of the manuscript. EL, JC, MH, KP, VN and MV drafted and revised the manuscript. All authors critically reviewed and approved the content of the manuscript. All authors had full access to all the data in the study and had final responsibility for the decision to submit for publication.

## Data sharing statement

The datasets generated from this review are available within the paper and Supplementary Materials. Details of any process, data or analysis are available from the corresponding author upon request.

## Declaration of interests

All other authors declare no competing interests.

## References

[1] Child mortality (under 5 years). https://www.who.int/news-room/fact-sheets/detail/levels-and-trends-in-child-under-5-mortality-in-2020.

[2] Kruk M.E., Gage A.D., Joseph N.T., Danaei G., García-Saisó S., Salomon J.A. (2018). Mortality due to low-quality health systems in the universal health coverage era: a systematic analysis of amenable deaths in 137 countries. Lancet.

[3] Rahmani A.M., Yousefpoor E., Yousefpoor M.S. (2021). Machine Learning (ML) in medicine: review, applications, and challenges. Mathematics.

[4] Fleuren L.M., Thoral P., Shillan D. Machine learning in intensive care medicine: ready for take-off? On behalf of the right data right now collaborators right data right now collaborators.

[5] van de Sande D., van Genderen M.E., Huiskens J., Gommers D., van Bommel J. (2021). Moving from bytes to bedside: a systematic review on the use of artificial intelligence in the intensive care unit. Intensive Care Med.

[6] Ezugwu A.E., Oyelade O.N., Ikotun A.M., Agushaka J.O., Ho Y.S. (2023). Machine learning research trends in Africa: a 30 years overview with bibliometric analysis review. Arch Comput Methods Eng.

[7] Onu C.C., Lebensold J., Hamilton W.L., Precup D. (2019). https://www.isca-archive.org/interspeech_2019/onu19_interspeech.html.

[8] Bellemo V., Lim Z.W., Lim G. (2019). Artificial intelligence using deep learning to screen for referable and vision-threatening diabetic retinopathy in Africa: a clinical validation study. Lancet Digit Health.

[9] Wahl B., Cossy-Gantner A., Germann S., Schwalbe N.R. (2018). Artificial intelligence (AI) and global health: how can AI contribute to health in resource-poor settings?. BMJ Glob Health.

[10] Hoodbhoy Z., Hasan B., Siddiqui K. (2019). Does artificial intelligence have any role in healthcare in low resource settings?. J Med Artif Intell.

[11] Williams D., Hornung H., Nadimpalli A., Peery A. (2021). Deep learning and its application for healthcare delivery in low and middle income countries. Front Artif Intell.

[12] Ahuja A.S., Schmidt C.E. (2019). The impact of artificial intelligence in medicine on the future role of the physician. PeerJ.

[13] Hornyak T. (2017). https://eos.org/articles/mapping-dengue-fever-hazard-with-machine-learning.

[14] Owoyemi A., Owoyemi J., Osiyemi A., Boyd A. (2020). Artificial intelligence for healthcare in Africa. Front Digit Health.

[15] Kondo T.S., Diwani S.A., Nyamawe A.S., Mjahidi M.M. (2025). Exploring the status of artificial intelligence for healthcare research in Africa: a bibliometric and thematic analysis. AI Ethics.

[16] Fantom N., Serajuddin U. (2016). https://openknowledge.worldbank.org/handle/10986/23628.

[17] World Bank Group Lower middle income | data. World Bank Group. https://data.worldbank.org/income-level/lower-middle-income.

[18] Zyl C.V., Badenhorst M., Hanekom S., Heine M. (2021). Unravelling ‘low-resource settings’: a systematic scoping review with qualitative content analysis. BMJ Glob Health.

[19] van Boven M.R., Henke C.E., Leemhuis A.G. (2022). Machine learning prediction models for neurodevelopmental outcome after preterm birth: a scoping review and new machine learning evaluation framework. Pediatrics.

[20] WHO (2025). https://platform.who.int/mortality/themes/theme-details/topics/topic-details/MDB/perinatal-conditions.

[21] Ouzzani M., Hammady H., Fedorowicz Z., Elmagarmid A. (2016). Rayyan-a web and mobile app for systematic reviews. Syst Rev.

[22] Page M., McKenzie J.E., Bossuyt P.M. (2021). The PRISMA 2020 statement: an updated guideline for reporting systematic reviews. BMJ.

[23] Moons K.G.M., Damen J.A.A., Kaul T. (2025). PROBAST+AI: an updated quality, risk of bias, and applicability assessment tool for prediction models using regression or artificial intelligence methods. BMJ.

[24] Annis A., Reaves C., Sender J., Bumpus S. (2023). Health-Related data sources accessible to health researchers from the US government: mapping review. J Med Internet Res.

[25] ACM (2025). https://dl.acm.org/ccs.

[26] Ahmed M.I., Spooner B., Isherwood J., Lane M., Orrock E., Dennison A. (2023). A systematic review of the barriers to the implementation of artificial intelligence in healthcare. Cureus.

[27] Innovative monitoring in paediatrics in low-resource settings: an aid to save lives?. ISRCTN Registry. https://www.isrctn.com/ISRCTN71392921.

[28] Babenko D., Seidullayeva A., Bayesheva D. (2021). Ability of procalcitonin and C-Reactive protein for discriminating between bacterial and enteroviral meningitis in children using decision tree. Biomed Res Int.

[29] Das S., Erdman L., Brals D. (2024). Development of machine learning models predicting mortality using routinely collected observational health data from 0-59 months old children admitted to an intensive care unit in Bangladesh: critical role of biochemistry and haematology data. BMJ Paediatr Open.

[30] Garbern S.C., Mamun G.M.S., Shaima S.N. (2024). A novel digital health approach to improving global pediatric sepsis care in Bangladesh using wearable technology and machine learning. PLOS Digit Health.

[31] Hsu J.F., Chang Y.F., Cheng H.J. (2021). Machine learning approaches to predict In-Hospital mortality among neonates with clinically suspected sepsis in the neonatal intensive care unit. J Pers Med.

[32] Hwang S., Lee B. (2022). Machine learning-based prediction of critical illness in children visiting the emergency department. PLoS One.

[33] Kanwal K., Khalid S.G., Asif M., Zafar F., Qurashi A.G. (2024). Diagnosis of Community-Acquired pneumonia in children using photoplethysmography and Machine learning-based classifier. Biomed Signal Process Control.

[34] Kashef A., Khatibi T., Mehrvar A. (2020). Prediction of cranial radiotherapy treatment in pediatric acute lymphoblastic leukemia patients using machine learning: a case study at MAHAK hospital. Asian Pac J Cancer Prev.

[35] Lee J.T., Hsieh C.C., Lin C.H., Lin Y.J., Kao C.Y. (2021). Prediction of hospitalization using artificial intelligence for urgent patients in the emergency department. Sci Rep.

[36] Lin S.R., Wu J.H., Liu Y.C. (2024). Machine learning models to evaluate mortality in pediatric patients with pneumonia in the intensive care unit. Pediatr Pulmonol.

[37] Liu H.F., Zhang X.Z., Liu C.Y. (2024). A novel combined nomogram for predicting severe acute lower respiratory tract infection in children hospitalized for RSV infection during the post-COVID-19 period. Front Immunol.

[38] Ming D.K., Hernandez B., Sangkaew S. (2022). Applied machine learning for the risk-stratification and clinical decision support of hospitalised patients with dengue in Vietnam. PLOS Digit Health.

[39] Oonsivilai M., Mo Y., Luangasanatip N. (2018). Using machine learning to guide targeted and locally-tailored empiric antibiotic prescribing in a children's hospital in Cambodia. Wellcome Open Res.

[40] Sheikhtaheri A., Zarkesh M.R., Moradi R., Kermani F. (2021). Prediction of neonatal deaths in NICUs: development and validation of machine learning models. BMC Med Inform Decis Mak.

[41] Tran P.N.T., Siranart N., Sukmark T. (2024). A simple nomogram to predict dengue shock syndrome: a study of 4522 South East Asian children. J Med Virol.

[42] Xue Y., Zhang J., Li C. (2022). Machine learning for screening and predicting the risk of anti-MDA5 antibody in juvenile dermatomyositis children. Front Immunol.

[43] Genisca A.E., Butler K., Gainey M. (2022). Constructing, validating, and updating machine learning models to predict survival in children with Ebola Virus Disease. PLoS Negl Trop Dis.

[44] Kovacs D., Msanga D.R., Mshana S.E., Bilal M., Oravcova K., Matthews L. (2021). Developing practical clinical tools for predicting neonatal mortality at a neonatal intensive care unit in Tanzania. BMC Pediatr.

[45] Kwizera A., Kissoon N., Musa N. (2019). A machine learning-based triage tool for children with acute infection in a low resource setting. Pediatr Crit Care Med.

[46] Pienaar M.A., Sempa J.B., Luwes N., Solomon L.J. (2022). An artificial neural network model for pediatric mortality prediction in two tertiary pediatric intensive care units in South Africa. A development study. Front Pediatr.

[47] Pienaar M.A., Sempa J.B., Luwes N., George E.C., Brown S.C. (2022). Development of artificial neural network models for paediatric critical illness in South Africa. Front Pediatr.

[48] Rahimi M., Wiens M.O., Kabakyenga J., Ansermino J.M., Dumont G.A. (2023). 2023 IEEE international conference on bioinformatics and biomedicine (BIBM).

[49] Robi Y.G., Sitote T.M. (2023). Neonatal disease prediction using machine learning techniques. J Healthc Eng.

[50] Domínguez-Rodríguez S., Serna-Pascual M., Oletto A. (2022). Machine learning outperformed logistic regression classification even with limit sample size: a model to predict pediatric HIV mortality and clinical progression to AIDS. PLoS One.

[51] Tunthanathip T., Duangsuwan J., Wattanakitrungroj N., Tongman S., Phuenpathom N. (2021). Comparison of intracranial injury predictability between machine learning algorithms and the nomogram in pediatric traumatic brain injury. Neurosurg Focus.

[52] Tuti T., Agweyu A., Mwaniki P., Peek N., English M., Clinical Information Network Author Group (2017). An exploration of mortality risk factors in non-severe pneumonia in children using clinical data from Kenya. BMC Med.

[53] Diallo A.H., Sayeem Bin Shahid A.S.M., Khan A.F. (2023). Characterising paediatric mortality during and after acute illness in Sub-Saharan Africa and South Asia: a secondary analysis of the CHAIN cohort using a machine learning approach. eClinicalMedicine.

[54] Rakers M., Mwale D., de Mare L. (2024). Cautiously optimistic: paediatric critical care nurses' perspectives on data-driven algorithms in low-resource settings—a human-centred design study in Malawi. BMC Glob Public Health.

[55] Austin P.C., Harrell F.E., Steyerberg E.W., Steyerberg E.W. (2021). Predictive performance of machine and statistical learning methods: impact of data-generating processes on external validity in the “large N, small p” setting. Stat Methods Med Res.

[56] Jing B., Boscardin W.J., Deardorff W.J. (2022). Comparing machine learning to regression methods for mortality prediction using veterans affairs electronic health record clinical data. Med Care.

[57] Rahman M.S., Islam K.R., Prithula J. (2024). Machine learning-based prognostic model for 30-day mortality prediction in Sepsis-3. BMC Med Inform Decis Mak.

[58] Rajula H.S.R., Verlato G., Manchia M., Antonucci N., Fanos V. (2020). Comparison of conventional statistical methods with machine learning in medicine: diagnosis, drug development, and treatment. Medicina.

[59] Abdulazeem H., Whitelaw S., Schauberger G., Klug S.J. (2023). A systematic review of clinical health conditions predicted by machine learning diagnostic and prognostic models trained or validated using real-world primary health care data. PLoS One.

[60] Jiang S., Dai S., Li Y. (2025). Development and validation of a screening tool for sepsis without laboratory results in the emergency department: a machine learning study. eClinicalMedicine.

[61] Frija G., Blažić I., Frush D.P. (2021). How to improve access to medical imaging in low- and middle-income countries?. eClinicalMedicine.

[62] Belge Bilgin G., Bilgin C., Burkett B.J. (2024). Theranostics and artificial intelligence: new frontiers in personalized medicine. Theranostics.

[63] Hoodbhoy Z., Masroor Jeelani S., Aziz A. (2021). Machine learning for child and adolescent health: a systematic review. Pediatrics.

[64] Ben-Israel D., Jacobs W.B., Casha S. (2020). The impact of machine learning on patient care: a systematic review. Artif Intell Med.

[65] Griesinger C.B., Reina V., Panidis D., Chassaigne H. (2025). https://data.europa.eu/doi/10.2760/8107037.

[66] Ashton J.J., Young A., Johnson M.J., Beattie R.M. (2023). Using machine learning to impact on long-term clinical care: principles, challenges, and practicalities. Pediatr Res.

[67] Faust L., Wilson P., Asai S. (2024). Considerations for quality control monitoring of machine learning models in clinical practice. JMIR Med Inform.

[68] Biana H.T., Joaquin J.J. (2025). The irony of AI in a low-to-middle-income country. AI Soc.

[69] Sandhu S., Lin A.L., Brajer N. (2020). Integrating a machine learning system into clinical workflows: qualitative study. J Med Internet Res.

[70] Yang H.S., Rhoads D.D., Sepulveda J., Zang C., Chadburn A., Wang F. (2022). Building the model: challenges and considerations of developing and implementing machine learning tools for clinical laboratory medicine practice. Arch Pathol Lab Med.

[71] Okolo C.T., Aruleba K., Obaido G., Eke D.O., Wakunuma K., Akintoye S. (2023). Responsible AI in Africa: challenges and opportunities.

[72] Rakers M., van de Vijver S., Bossio P. (2023). SERIES: eHealth in primary care. Part 6: global perspectives: learning from eHealth for low-resource primary care settings and across high-middle- and low-income countries. Eur J Gen Pract.

[73] Ethics guidelines for trustworthy AI | Shaping Europe's digital future. https://digital-strategy.ec.europa.eu/en/library/ethics-guidelines-trustworthy-ai.

[74] Eke D.O., Wakunuma K., Akintoye S., Ogoh G. (2025). Trustworthy AI: African perspectives.

[75] Khan M.S., Umer H., Faruqe F. (2024). Artificial intelligence for low income countries. Humanit Soc Sci Commun.

[76] Cisse M. (2018). Look to Africa to advance artificial intelligence. Nature.

[77] Rakers M.M., van Buchem M.M., Kucenko S. (2024). Availability of evidence for predictive machine learning algorithms in primary care: a systematic review. JAMA Netw Open.

