# Supplementary material for: Machine learning for predicting clinical outcomes of hospitalised children: a systematic review of applications in low- and middle-income countries
Source: eClinicalMedicine. 2026 Jan 8;91:103743. doi: 10.1016/j.eclinm.2025.103743 (PMC12818084; doi:10.1016/j.eclinm.2025.103743)
Supplement: Supplementary Appendix [file mmc1.docx]

**Appendix A. Supplementary data**

**Machine Learning for Predicting Clinical Outcomes of Hospitalised Children: A Systematic Review of Applications in Low- and Middle-Income Countries**

William Nkhono, Eva van Lieshout, Job Calis, Violet Naanyu, Mark Hoogendoorn, Kamija S Phiri, María Villalobos-Quesada.

<https://doi.org/10.1016/j.eclinm.2025.103743>

Table of Contents

[Prisma Checklist 3](#_Toc217882719)

[Search strategy 5](#_Toc217882720)

[PubMed 5](#_Toc217882721)

[Google Scholar 5](#_Toc217882722)

[IEEE Xplore 5](#_Toc217882723)

[Web of Science 5](#_Toc217882724)

[Embase 5](#_Toc217882725)

[Scopus 5](#_Toc217882726)

[Table S1: Summary of Study characteristics (PICOTS*) 8](#_Toc217882727)

[Table S2: Classification of prediction model studies (PROBAST+AI) 9](#_Toc217882728)

[Table S3: Summary of assessment of quality and concern about applicability of PROBAST+AI 10](#_Toc217882729)

[Table S4: Summary of assessment of risk of bias and concern about applicability of PROBAST+AI 11](#_Toc217882730)

[Figure S1: Summary of assessment of Quality, Risk of Bias and Applicability Concerns by PROBAST+AI Domain 12](#_Toc217882731)

# Prisma Checklist

| **Section and Topic** | **Item #** | **Checklist item** | **Location where item is reported** |
| --- | --- | --- | --- |
| **TITLE** | | |  |
| Title | 1 | Identify the report as a systematic review. | Page 1 |
| **ABSTRACT** | | |  |
| Abstract | 2 | See the PRISMA 2020 for Abstracts checklist. | Page 1 |
| **INTRODUCTION** | | |  |
| Rationale | 3 | Describe the rationale for the review in the context of existing knowledge. | Page 3 |
| Objectives | 4 | Provide an explicit statement of the objective(s) or question(s) the review addresses. | Page 3 |
| **METHODS** | | |  |
| Eligibility criteria | 5 | Specify the inclusion and exclusion criteria for the review and how studies were grouped for the syntheses. | Page 4 |
| Information sources | 6 | Specify all databases, registers, websites, organisations, reference lists and other sources searched or consulted to identify studies. Specify the date when each source was last searched or consulted. | Page 3 |
| Search strategy | 7 | Present the full search strategies for all databases, registers and websites, including any filters and limits used. | Page 4 |
| Selection process | 8 | Specify the methods used to decide whether a study met the inclusion criteria of the review, including how many reviewers screened each record and each report retrieved, whether they worked independently, and if applicable, details of automation tools used in the process. | Page 4 |
| Data collection process | 9 | Specify the methods used to collect data from reports, including how many reviewers collected data from each report, whether they worked independently, any processes for obtaining or confirming data from study investigators, and if applicable, details of automation tools used in the process. | Page 4 |
| Data items | 10a | List and define all outcomes for which data were sought. Specify whether all results that were compatible with each outcome domain in each study were sought (e.g. for all measures, time points, analyses), and if not, the methods used to decide which results to collect. | Page 4 |
|  | 10b | List and define all other variables for which data were sought (e.g. participant and intervention characteristics, funding sources). Describe any assumptions made about any missing or unclear information. | Page 4 |
| Study risk of bias assessment | 11 | Specify the methods used to assess risk of bias in the included studies, including details of the tool(s) used, how many reviewers assessed each study and whether they worked independently, and if applicable, details of automation tools used in the process. | Page 4 |
| Effect measures | 12 | Specify for each outcome the effect measure(s) (e.g. risk ratio, mean difference) used in the synthesis or presentation of results. | Page 6 |
| Synthesis methods | 13a | Describe the processes used to decide which studies were eligible for each synthesis (e.g. tabulating the study intervention characteristics and comparing against the planned groups for each synthesis (item #5)). | Page 5 |
|  | 13b | Describe any methods required to prepare the data for presentation or synthesis, such as handling of missing summary statistics, or data conversions. | Page 5 |
|  | 13c | Describe any methods used to tabulate or visually display results of individual studies and syntheses. | Page 5 |
|  | 13d | Describe any methods used to synthesize results and provide a rationale for the choice(s). If meta-analysis was performed, describe the model(s), method(s) to identify the presence and extent of statistical heterogeneity, and software package(s) used. | Page 5 |
|  | 13e | Describe any methods used to explore possible causes of heterogeneity among study results (e.g. subgroup analysis, meta-regression). | Page 5 |
|  | 13f | Describe any sensitivity analyses conducted to assess robustness of the synthesized results. | Page 5 |
| Reporting bias assessment | 14 | Describe any methods used to assess risk of bias due to missing results in a synthesis (arising from reporting biases). | Page 6 |
| Certainty assessment | 15 | Describe any methods used to assess certainty (or confidence) in the body of evidence for an outcome. | Page 5 |
| **RESULTS** | | |  |
| Study selection | 16a | Describe the results of the search and selection process, from the number of records identified in the search to the number of studies included in the review, ideally using a flow diagram. | Page 5 |
|  | 16b | Cite studies that might appear to meet the inclusion criteria, but which were excluded, and explain why they were excluded. | Page 5 |
| Study characteristics | 17 | Cite each included study and present its characteristics. | Page 5 to 8 |
| Risk of bias in studies | 18 | Present assessments of risk of bias for each included study. | Page 8 |
| Results of individual studies | 19 | For all outcomes, present, for each study: (a) summary statistics for each group (where appropriate) and (b) an effect estimate and its precision (e.g. confidence/credible interval), ideally using structured tables or plots. | Page 5 to 8 |
| Results of syntheses | 20a | For each synthesis, briefly summarise the characteristics and risk of bias among contributing studies. | Page 8 |
|  | 20b | Present results of all statistical syntheses conducted. If meta-analysis was done, present for each the summary estimate and its precision (e.g. confidence/credible interval) and measures of statistical heterogeneity. If comparing groups, describe the direction of the effect. | Page 5 to 8 |
|  | 20c | Present results of all investigations of possible causes of heterogeneity among study results. | Page 5 to 8 |
|  | 20d | Present results of all sensitivity analyses conducted to assess the robustness of the synthesized results. | Page 5 to 8 |
| Reporting biases | 21 | Present assessments of risk of bias due to missing results (arising from reporting biases) for each synthesis assessed. | Page 8 |
| Certainty of evidence | 22 | Present assessments of certainty (or confidence) in the body of evidence for each outcome assessed. | Page 5 to 8 |
| **DISCUSSION** | | |  |
| Discussion | 23a | Provide a general interpretation of the results in the context of other evidence. | Page 8 |
|  | 23b | Discuss any limitations of the evidence included in the review. | Page 9 |
|  | 23c | Discuss any limitations of the review processes used. | Page 10 |
|  | 23d | Discuss implications of the results for practice, policy, and future research. | Page 9 |
| **OTHER INFORMATION** | | |  |
| Registration and protocol | 24a | Provide registration information for the review, including register name and registration number, or state that the review was not registered. | Page 6 |
|  | 24b | Indicate where the review protocol can be accessed, or state that a protocol was not prepared. | NA |
|  | 24c | Describe and explain any amendments to information provided at registration or in the protocol. | NA |
| Support | 25 | Describe sources of financial or non-financial support for the review, and the role of the funders or sponsors in the review. | Page 14 |
| Competing interests | 26 | Declare any competing interests of review authors. | Page 14 |
| Availability of data, code and other materials | 27 | Report which of the following are publicly available and where they can be found: template data collection forms; data extracted from included studies; data used for all analyses; analytic code; any other materials used in the review. | Page 14 |

# Search strategy

## PubMed


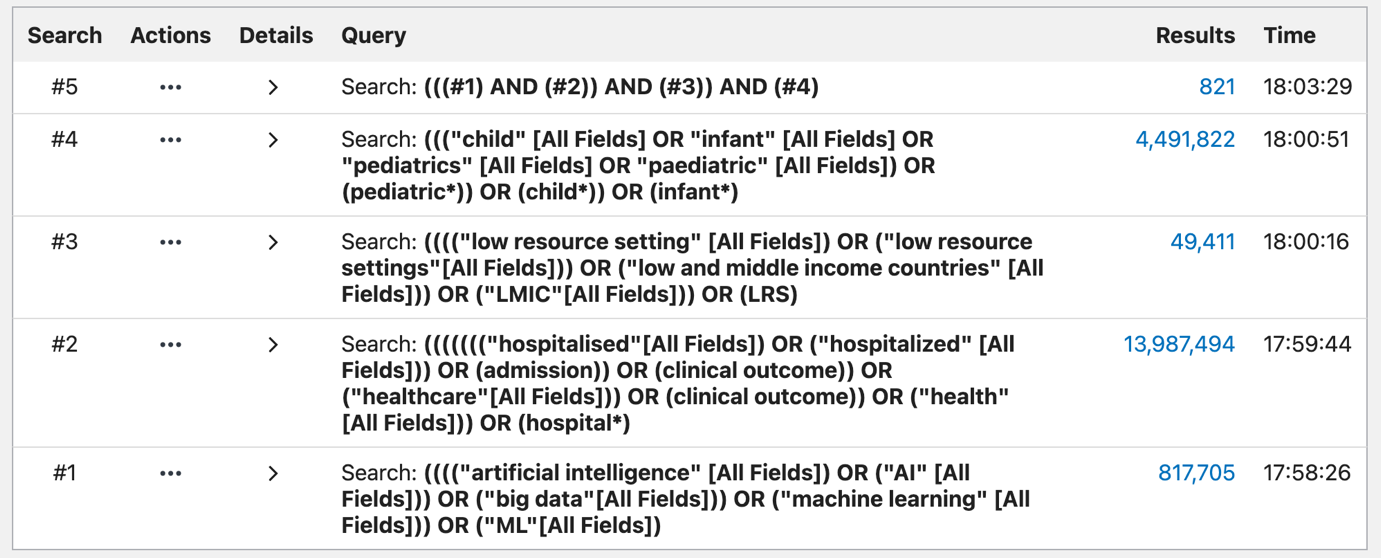


## Google Scholar

(“artificial intelligence” OR “machine learning”) (“hospitalised” OR “admission” OR “clinical outcome”) (child* OR infant OR paediatric OR pediatric) (“low resource setting” OR “low and middle income countries”).

## IEEE Xplore

(“All Metadata”:“artificial intelligence” OR “All Metadata”:“machine learning”) AND (“All Metadata”:hospitalised OR “All Metadata”:admission OR “All Metadata”:clinical outcome) AND (“All Metadata”:child OR “All Metadata”:infant OR “All Metadata”:paediatric) AND (“All Metadata”:low resource setting OR “All Metadata”: low and middle-income countries)

## Web of Science

(((ALL=(“artificial intelligence” OR “machine learning”)) AND ALL=( hospitalised OR admission OR “clinical outcome”)) AND ALL=( child OR infant OR paediatric OR pediatric)) AND ALL=(“low resource setting” OR “low middle-income countries”)

## Embase

('artificial intelligence' OR 'machine learning') AND ('hospital admission' OR 'clinical outcome') AND ('child' OR 'infant' OR 'pediatrics') AND ('low resource setting' OR ' low and middle income countries ' OR 'low income country')

## Scopus

( TITLE-ABS-KEY ( artificial intelligence ) OR TITLE-ABS-KEY ( machine learning ) AND TITLE-ABS-KEY ( hospital admission ) OR TITLE-ABS-KEY ( clinical outcome ) AND TITLE-ABS-KEY ( child ) OR TITLE-ABS-KEY ( infant ) OR TITLE-ABS-KEY ( pediatric ) AND TITLE-ABS-KEY ( low resource setting ) OR TITLE-ABS-KEY ( low and middle income countries) OR TITLE-ABS-KEY ( low income countries )

| **Author** | **Population** | **Index model(s)** | **Outcome(s)** | **Timing** | **Setting** |
| --- | --- | --- | --- | --- | --- |
| Babenko | Children aged 1 month-17 years with meningitis in Kazakhstan. | Fast-and-Frugal Trees decision tree. | Meningitis | At patient presentation, using clinical and lab results. | Single tertiary children's hospital in Kazakhstan. |
| CHAIN network | Children aged 2-24 months hospitalized for acute illness in SSA and South Asia. | XGBoost Cox proportional hazards models. | Mortality | At admission and at discharge. | Nine hospitals in LMICs. |
| Das | Children aged 0-59 months admitted to an ICU in Bangladesh. | Random Forest (RF) model | Mortality | At admission to ICU. | ICU in Dhaka, Bangladesh |
| Domínguez-Rodríguez | Infants with perinatally acquired HIV from South Africa and Mozambique. | Random Forest (RF). | Mortality | At enrollment (after ART initiation). | Multicentre study in South Africa & Mozambique. Predict outcomes for staff in limited resource settings. |
| Garbern | Children aged 2 months-17 years with suspected sepsis, admitted to the ICU in Dhaka, Bangladesh. | Ridge regression models (Model B & C). | Sepsis | Continuous from admission, for real-time prediction. | ICU in Dhaka, Bangladesh. |
| Genisca | Children aged <18 years with laboratory-confirmed EVD. | Elastic Net model. | Mortality | At admission to an ETU, using data from the first 48 hours. | ETUs in West Africa & DRC. Bedside tool for resource allocation. |
| Hsu | All neonates hospitalized in the NICUs of Chang Gung Memorial Hospital (CGMH). | Deep Neural Network, K-nearest neighbors, support vector machine, random forest, extreme gradient boost, Glmnet, and regression tree algorithm (Treebag). | Mortality | Onset of the first unstable episode of clinically suspected sepsis, in hospital mortality (prior to discharge). | Single-center study at the NICU. |
| Hwang | Children aged <15 years visiting EDs in South Korea. | Random Forest (RF) classification model. | Critical illness | At initial triage in the ED. | Nationwide registry (400+ EDs) in South Korea. Augment conventional triage process. |
| Kanwal | Paediatric participants with CAP or healthy controls. | Weighted KNN, Ensemble of Bagged Trees. | Diagnosis of Community-Acquired Pneumonia | At the time of PPG recording. | PICU in Karachi, Pakistan. Non-invasive, low-cost screening in low-resource settings. |
| Kashef | Pediatric Acute Lymphoblastic Leukemia patients aged 3 months to 17 years treated at Mahak Charity Hospital in Tehran. | stacked ensemble classifier compared against Individual base models DRF, GBM, Generalized Linear Model. | Cranial radiotherapy | At the time of diagnosis or during the course of therapy. | Mahak Charity Hospital, a specialized center in Iran. |
| Kovacs | Neonates admitted to NICU in Mwanza, Tanzania. | Generalised Linear Models (GLMs). | Mortality | At NICU admission, using first measurements. | NICU in Tanzania. Easy-to-use graphical tool for early warning. |
| Kwizera | Children (28 days-18 years) hospitalized for acute infection in Rwanda. | Random Forest (RF) model. | Mortality | At hospital admission. | Rural hospital in Rwanda. Triage tool for optimizing resource allocation. |
| Lee | Patients visiting the Emergency Department (ED). | A three-layer neural network model developed to predict hospital admission. | Hospitalisation | At initial triage in the ED, using data readily available in most ED triage systems. | ED of a tertiary teaching hospital in Tainan city. |
| Lin | Children aged <18 years with pneumonia admitted to PICU in Taiwan. | Random Forest (RF), XGBoost (XGB). | Mortality | At ICU admission (first 24h) and daily thereafter. | Single tertiary hospital in Taiwan. Aid decision-making in resource-limited settings. |
| Liu | Children (≤14 years) hospitalized for RSV infection in Yunnan, China. | Nomogram model based on Logistic Regression. | Severe acute lower respiratory tract infection | At hospital admission. | 9 tertiary hospitals in China. Prediction tool to aid clinical decision-making. |
| Ming | Hospitalised adults and children. | ANN, XGBoost, Random Forest, Support Vector Machine, Logistic Regression. | Dengue shock syndrome | Predicts within 48h of admission, for outcome during hospitalization. | Tertiary hospital in Vietnam. A clinical decision support system. |
| Oonsivilai | Children (<16 years) with bloodstream infections in Cambodia. | Random Forest (RF). | Antibiotic susceptibility | At blood culture collection, for immediate prediction. | Children's hospital in Cambodia. Decision support for empiric antibiotic therapy. |
| Pienaar | Patients up to the age of 13 years admitted to the Pediatric Intensive Care Units (PICUs) of Pelonomi Tertiary Hospital and Universitas Tertiary Hospital. | Artificial Neural Network (ANN) models. A Logistic Regression (LR) model developed on the same data set, and a recalibrated version of the Pediatric Index of Mortality 3 (PIM3). | Mortality | Within the first hour of admission to the PICU (based on data collected for PIM3 scores). | Two tertiary PICUs in South Africa (Pelonomi and Universitas Hospitals). |
| Pienaar | Children under the age of 13 years presenting for unscheduled consultations (duration of illness/injury < 7 days, including acute exacerbations) to the Paediatric Referral Area of Pelonomi Regional Hospital. | Artificial Neural Network (ANN). Logistic Regression (LR) and XGBoost (XGB) models. | Mortality | At initial assessment in the Paediatric Referral Area. | A single tertiary hospital in South Africa (Pelonomi Regional Hospital). |
| Rahimi | Two cohorts of children hospitalized with confirmed or suspected infectious illnesses in Uganda. | Five supervised machine learning algorithms were tested, with the Balanced Random Forest Classifier (BRFC) identified as the superior performer. | Mortality | At hospital admission using admission-recorded information. | A multi-site, observational cohort study in Uganda. |
| Robi | Neonates admitted to the NICU of Asella Comprehensive Hospital. | A classification stacking model compared to Individual models: XGBoost (XGB), Random Forest (RF), and Support Vector Machine (SVM). | Sepsis, birth asphyxia, necrotizing enterocolitis, and respiratory distress syndrome | At admission to the NICU, using data collected from patient cards (admission information, delivery information, symptoms, laboratory results, and X-ray results). | NICU of Asella Comprehensive Hospital. |
| Sheikhtaheri | Neonates admitted to NICUs in Tehran, Iran. | ANN, RF, SVM, etc. (Ensemble/Stacking models). | Mortality | At admission to NICU. | Teaching hospitals in Iran. Aid physicians in predicting neonatal deaths. |
| Tran | cohort of 4522 children with confirmed dengue infection admitted to King Chulalongkorn Memorial Hospital in Thailand. | A simple nomogram based on a Logistic Regression model. Alternative machine learning algorithms were compared, including Random Forest (RF), XGBoost (XGB), and Support Vector Machine (SVM). | Dengue shock syndrome | At hospital admission/enrollment. | Pooled analysis of cohorts from two major hospitals in Southeast Asia. |
| Tunthanathip | Children aged <15 years) with TBI in Hat Yai, Thailand. | RFC, SVM, LR, etc. | Intracranial injury | At presentation, before CT is performed. | Single neurosurgical center in Thailand. Guide decisions on CT use. |
| Tuti | Children aged 2–59 months. | Machine learning models, including Partial Least Squares Discriminant Analysis (PLS-DA), Random Forests (RFs), Support Vector Machines (SVMs), and Elastic Nets. The study highlights PLS-DA for its high sensitivity. | Mortality | At admission to the hospital, using predictors assessed at that moment. | 14 public hospitals in Kenya, selected to represent different regional malaria prevalences. |
| Xue | Patients with Juvenile Dermatomyositis (JDM). | A Random Forest (RF) algorithm was chosen to predict anti-MDA5 antibody positivity. Stepwise logistic regression (SLR) and LASSO logistic regression. | Screening anti-MDA5 antibodies | At the time of JDM diagnosis. | Single-center study at Beijing Children’s Hospital, China. The tool is intended to screen for anti-MDA5 antibodies in JDM patients. |

# Table S1: Summary of Study characteristics (PICOTS*) Population, Index/comparator, Outcome, Timing of prediction and of outcome and Setting

| **Author** | **Prediction model study** | **Comment** |
| --- | --- | --- |
| Babenko | Development only (with Internal Validation) | The study developed a new model and evaluated its performance using a split-sample approach (80% training, 20% testing) within the same cohort. |
| CHAIN network | Combination (Development and Evaluation) | The study developed new models and performed internal validation using a repeated train-test split. |
| Das | Development only (with Internal Validation) | The study developed new machine learning models and performed internal validation using 10-fold cross-validation and a held-out test set. |
| Domínguez-Rodríguez | Development only (with Internal Validation) | The study developed new machine learning models and performed internal validation using a random split and repeated cross-validation. |
| Garbern | Combination (Development and Evaluation) | The study developed new machine learning models and performed internal validation using 5-fold cross-validation. |
| Genisca | Combination (Development and Evaluation) | The study derived a new model, performed internal validation, and then conducted external validation and model updating using a distinct dataset. |
| Hsu | Development only (with Internal Validation) | Performs internal validation using a random split into a training and a test set. |
| Hwang | Development only (with Internal Validation) | The study developed a new RF model and performed internal validation using 5-fold cross-validation on an under-sampled dataset, then validated on the entire dataset. |
| Kanwal | Development only (with Internal Validation) | The study developed new machine learning classifiers and performed internal validation using 5-fold cross-validation and a separate test set. |
| Kashef | Development only (with Internal Validation) | The study developed a new model and performed internal validation using a train-test split and cross-validation within the same dataset. |
| Kovacs | Development only (with Internal Validation) | The study developed new predictive models and performed internal validation by resampling the data to create training and test datasets. |
| Kwizera | Development only (with Internal Validation) | The study developed new Random Forest models and performed internal validation using a 5-fold cross-validation procedure. |
| Lee | Development only (with Internal Validation) | The study developed a model to predict hospitalization using a neural network and evaluated its performance on a held-out test set. |
| Lin | Development only (with Internal Validation) | The study developed new machine learning models and performed internal validation using a time-based split and cross-validation. |
| Liu | Combination (Development and Evaluation) | The study developed a new nomogram model, performed internal validation, and then conducted external validation using independent cohorts. |
| Ming | Combination (Development and Evaluation) | The study developed new models and performed internal cross-validation and evaluation on an independent hold-out set. |
| Oonsivilai | Development only (with Internal Validation) | The study developed new machine learning models and performed internal validation using 5-fold cross-validation and repeated train-test splits. |
| Pienaar | Development only (with Internal Validation) | The study developed new Artificial Neural Network models and performed internal validation using a train-test split and cross-validation. |
| Pienaar | Development only (with Internal Validation) | The study developed new models and evaluated their performance on a held-out test set using cross-validation. |
| Rahimi | Development only (with Internal Validation) | The study developed new predictive models and performed internal validation using a held-out test set and bootstrapping. |
| Robi | Development only (with Internal Validation) | The study developed a new stacking model and performed internal validation using stratified k-fold cross-validation. |
| Sheikhtaheri | Combination (Development and Evaluation) | The study developed new machine learning models using retrospective data and conducted prospective external validation on a new dataset. |
| Tran | Development only (with Internal Validation) | The study developed a new nomogram model and performed internal validation by splitting a single cohort into training, testing, and validating sets. |
| Tunthanathip | Development only (with Internal Validation) | The study developed new machine learning algorithms and a nomogram, performing internal validation using a random split and cross-validation. |
| Tuti | Development only (with Internal Validation) | The study developed new predictive models and performed internal validation using a hold-out set and cross-validation. |
| Xue | Development only (with Internal Validation) | The study developed a new prediction model and performed internal validation using 500 bootstrapped replications. |

# Table S2: Classification of prediction model studies (PROBAST+AI)

| **Author** | **D1: (Q/A) Participants & Data Sources** | **D2: Predictors (Q/A)** | **D3: Outcome (Q/A)** | **D4: Analysis** |
| --- | --- | --- | --- | --- |
| Babenko | High / Low | Low / Low | Low / Low | High |
| CHAIN network | High / Low | High / Unc | Low / Low | Low |
| Das | High / Low | High / Unc | Low / Low | High |
| Domíngez-Rodriguez | High / Low | Low / Low | Low / Low | High |
| Garbern | High / Low | Low / Unc | Low / Low | High |
| Genisca | High / Low | High / Low | Low / Low | Low |
| Hsu | High / Low | Low / Low | Low / Low | High |
| Hwang | High / Low | Low / Low | High / Low | High |
| Kanwal | High / Unc | Low / Low | High / Low | High |
| Kashef | High / Low | High / Low | High / Low | High |
| Kovacs | High / Low | Low / Low | Low / Low | High |
| Kwizera | High / Low | Low / Low | Low / Low | High |
| Lee | High / Low | Low / Low | Low / Low | High |
| Lin | High / Low | Low / Low | Low / Low | High |
| Liu | Low / Low | Low / Low | Low / Low | Low |
| Ming | High / Low | Low / Low | Low / Low | Low |
| Oonsivilai | High / Low | Low / Low | Low / Low | High |
| Pienaar (1) | High / Low | Low / Low | Low / Low | Low |
| Pienaar (2) | Low / Low | Low / Low | Low / Low | Low |
| Rahimi | High / Low | Low / Low | Low / Low | High |
| Robi | High / Low | High / Low | High / Low | High |
| Sheikhtaheri | High / Low | High / Low | Low / Low | High |
| Tran | High / Low | Low / Low | Low / Low | Low |
| Tunthanathip | High / Low | High / Low | Low / Low | High |
| Tuti | High / Low | Low / Low | Low / Low | High |
| Xue | High / Unc | Low / Low | Low / Low | High |

# Table S3: Summary of assessment of quality and concern about applicability of PROBAST+AI, Q = Quality; A = Applicability; Unc = Unclear; High = High Concern; Low = Low Concern

| **Author** | **D1: (RB/A) Participants & Data Sources** | **D2: Predictors (RB/A)** | **D3: Outcome (RB/A)** | **D4: Analysis** |
| --- | --- | --- | --- | --- |
| Babenko | High/Low | Low/Low | Low/Low | High |
| CHAIN network | High/Low | Low/Unc | Low/Low | High |
| Das | High/Low | Low/Unc | Low/Low | High |
| Domíngez-Rodríguez | High/Low | Low/Low | Low/Low | High |
| Garbern | High/Low | Low/Unc | Low/Low | High |
| Genisca | High/Low | Low/Low | Low/Low | High |
| Hsu | High/Low | Low/Low | Low/Low | High |
| Hwang | Low/Low | Low/Low | Low/Low | High |
| Kanwal | High/Unc | Low/Low | High/Low | High |
| Kashef | High/Low | High/Low | High/Low | High |
| Kovacs | High/Low | Low/Low | Low/Low | High |
| Kwizera | High/Low | Low/Low | Low/Low | High |
| Lee | High/Low | Low/Low | Low/Low | High |
| Lin | High/Low | Low/Low | Low/Low | High |
| Liu | Low/Low | Low/Low | Low/Low | Low |
| Ming | High/Low | Low/Low | Low/Low | Low |
| Oonsivilai | High/Low | Low/Low | Low/Low | High |
| Pienaar (1) | High/Low | Low/Low | Low/Low | Low |
| Pienaar (2) | High/Low | Low/Low | Low/Low | High |
| Rahimi | High/Low | Low/Low | Low/Low | High |
| Robi | High/Low | High/Low | High/Low | High |
| Sheikhtaheri | High/Low | High/Low | Low/Low | High |
| Tran | High/Low | Low/Low | Low/Low | Low |
| Tunthanathip | High/Low | High/Low | Low/Low | High |
| Tuti | High/Low | Low/Low | Low/Low | High |
| Xue | High/Unc | Low/Low | Low/Low | High |

# Table S4: Summary of assessment of risk of bias and concern about applicability of PROBAST+AI; Q = Quality; A = Applicability; Unc = Unclear; High = High concern, Low = Low concern


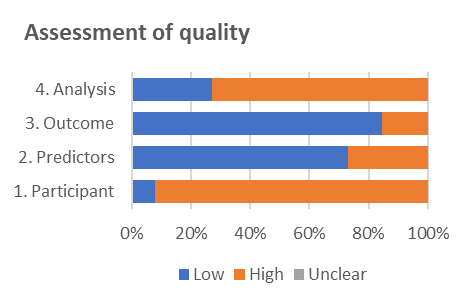

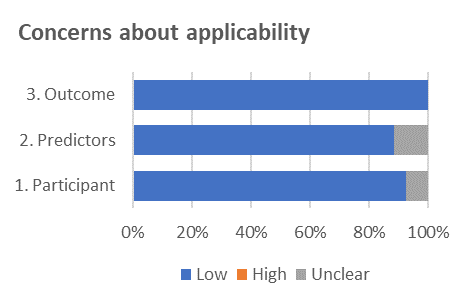

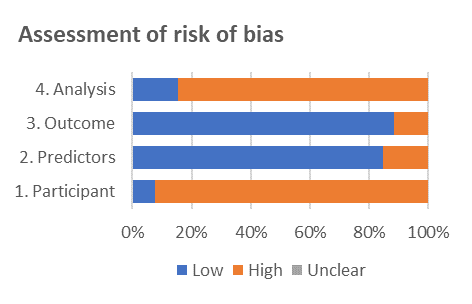

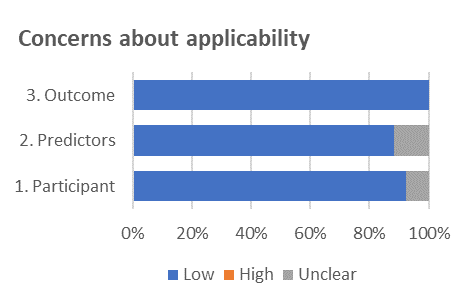


# Figure S1: Summary of assessment of Quality, Risk of Bias and Applicability Concerns by PROBAST+AI Domain

The PROBAST+AI assessment highlighted a high concern regarding methodological rigour in prediction models for LMICs. Weaknesses in development, such as small sample sizes, retrospective study design, heterogeneous data sources and selection bias, reduced model quality and generalizability.

Risk of bias during evaluation highlighted a high concern due to the absence of a formal calibration assessment and net benefit reported in 6 studies (23%). Most models showed a strong discrimination with a mean AUC of 0.80 (IQR 0.78 – 0.83), but a few reported other evaluation metrics, such as positive predictive values (PPV), suggesting frequent false alarms. The limited evaluation metrics limited clinical applicability.

Applicability concerns were low, as most models were developed and tested in relevant populations and settings that represented their intended clinical use. However, some studies relied on predictors not universally available in LMICs, such as advanced laboratory tests.

While most models perform well and discriminate outcomes, systematic methodological rigour, including poor data foundations, incomplete performance evaluation and risk of bias, weakens their trustworthiness and real-world utility.
